# Health-related quality of life and its determinants among ambulatory patients with epilepsy at Ambo General Hospital, Ethiopia: Using WHOQOL-BREF

**DOI:** 10.1371/journal.pone.0227858

**Published:** 2020-01-21

**Authors:** Gosaye Mekonen Tefera, Worku Asefa Megersa, Diriba Alemayehu Gadisa

**Affiliations:** 1 Department of Pharmacy, Clinical Pharmacy Unit, Ambo University, Ambo, Ethiopia; 2 Department of Pharmacy, Pharmacology Unit, Ambo University, Ambo, Ethiopia; Qazvin University of Medical Sciences, ISLAMIC REPUBLIC OF IRAN

## Abstract

**Background:**

Health‐related quality of life (HRQOL) is used as a measure of treatment outcomes, in addition to seizure control. Hence, the study was aimed to assess HRQOL and its determinants among adult patients with epilepsy (PWE).

**Method:**

A hospital-based cross-sectional study was conducted from April 20 to June 27/2019, through patient interviews and patient’s chart review (medication and clinical information). Data were analyzed using SPSS version 20. The psychometric property was done using Cronbach’s alpha test value of >0.7 as accepted internal consistency and Kaiser-Meyer-Olkin (KMO) measure of sample size adequacy value of ≥0.5 as acceptable construct validity, for Afaan Oromo version questionnaire. Multivariate linear logistic regression analysis was done to find predictors for the HRQOL score.

**Results:**

Of 121 PWE included in the study, 24.4% had overall poor HRQOL with the overall mean ±SD score of 56.42±10.96. The predictors for low overall HRQOL score were: presence of co-morbidity (B = -5.620, SE = 1.531, p<0.0001, 95% C.I = -8.656 to -2.584), uncontrolled seizure for at least 2 years (B = -4.239, SE = 1.296, p = 0.001, 95% C.I = -6.809 to -1.670), divorced (B = -8.423, SE = 2.241, p<0.0001, 95%C.I = -12.867 to -3.978) relative to married, and no education (B = -8.715, SE = -8.15, SE = 2.604, p = 0.001, 95%C.I = -9.648 to -1.316) relative to who had level of education above tertiary. In addition, uncontrolled seizure (irrespective of time since seizure-free) (B = -10.083, SE = 2.104, p<0.0001, 95%C.I = -14.256 to -5.910), being widowed (B = -9.300, SE = 3.594, p = 0.011, 95%C.I = -16.429–2.170) relative to married and being illiterate/no education (B = -13.004, SE = 3.910, p = 0.001, 95%C.I = -20.760 to -5.248) relative to educational level of tertiary and above were found to be the strongest negative predictors of HRQOL of physical health. Moreover, uncontrolled seizure (irrespective of time since seizure-free) (B = -12.668, SE = 2.019, p<0.0001, 95%C.I = -16.671 to -8.664) and being divorced (B = -10.153, SE = 3.228, p = 0.002, 95%C.I = -16.556 to -3.751) compared to married were strong predictors for low HRQOL score of psychological health. Absence of Poly-pharmacy (B = 9.050, SE = 3.027, P = 0.003, 95%C.I = 3.047 to 15.054), being single (B = -9.551, SE = 2.095, p<0.0001, 95%C.I = -14.419 to -4.683), and divorced (B = -11.022, SE = 3.351, P = 0.001, 95%C.I = -17.668 to -4.376) relative to married were found to be strong predictors for HRQOL score of social health. Moreover, low HRQOL score of environmental health was predicted by rural residence (B = -5.795, SE = 2.101, p = 0.007, 95%C.I = -9.962 to -1.628), co-morbidity (B+ -4.230, SE = 2.125, p = 0.049, 95%C.I = -8.444 to -0.015) & uncontrolled seizure irrespective of time since seizure-free (B = -6.907, SE = 1.945, p = 0.001, 95%C.I = -10.765 to -3.049) and uncontrolled seizure of at least 2 years (B = -4.520, SE = 1.798, p = 0.014, 95%C.I = -8.088 to -0.953).

**Conclusions:**

The majority of the study participants had a good overall HRQOL. In general, a low level of HRQOL score was significantly associated with the marital status of single/widowed/divorced, low level of education, low level of monthly income, co-morbidity, uncontrolled seizure, and poly-pharmacy; irrespective of HRQOL domains. Therefore, it is required to improve HRQOL, by avoiding modifiable factors for PWE to achieve the optimum HRQOL.

## Introduction

Epilepsy is a non-communicable disease of the brain that is characterized by two or more unprovoked seizures occurring more than 24 hours apart or one unprovoked seizure when the risk for another is known to be high (>60%)[1]. The International League against Epilepsy (ILAE) classifies seizures according to either the clinical presentation or the underlying pathology into three major types of seizure (focal seizures, generalized seizures, and unclassified seizures) [1, 2].

Approximately more than 50 to 70 million people worldwide have epilepsy, making it one of the most common neurological diseases globally [3–5].Epilepsy is one of the non-communicable global diseases with an unequal distribution, in which 80% to 90% of the affected individuals reside in low and middle-income countries (LMIC) [3–9]. Besides this, six-fold higher mortality in LMIC than high-income countries[10].The high incidence and prevalence of epilepsy in LMICs are to some extent explained by more exposure to predisposing factors; especially in rural areas of LMICs than that of higher-income countries[5, 6, 9]. The 2003 world health organization (WHO) data suggest that epilepsy is among the most important contributors to the global burden of human suffering. As a result WHO and other stakeholders regard epilepsy as a priority, especially in LMIC [6, 11].

Epilepsy is considered a treatable condition with high rates of therapeutic response (75%) with the use of currently available antiepileptic drugs (AED). However, the treatment gap (TG) in LMICs remains very high (75–90%) [6, 8, 12]. TG in Ethiopia was even higher than other LMIC; which is 98.7% in rural parts of the country and as high as 87% in Addis Ababa[13].

Epilepsy has serious physical, psychological, social and socio-economic consequences for the concerned persons and their families[12]; owing to stigma and discrimination from the community [3]. Because many people in Africa believe that epilepsy is contagious. Hence, during convulsion peoples are unwilling to help or to touch the person who has fallen during a seizure (lack of support during an emergency from the community)[1, 2]. Due to earlier mentioned reason and the need for regular medications, their side effects and also due to prejudices and social conventions that surround it, epilepsy can affect the Health-related Quality of Life (HRQOL) negatively[14]. Furthermore, the appearance of psychosocial and medical barriers that promote less opportunity for education, lower school performance, less employment, lower annual income, low self-esteem, feelings of shame and guilt, and less marriage which hinders the involvement of patients with epilepsy (PWE) in full social life compared with individuals without epilepsy [6, 15–18].

Thus epilepsy is known to hijack the lives of PWE and their families; hence PWE had poor HRQOL[17, 19, 20] and they are the most vulnerable society because of misconception and stigma from their community[9, 17, 21].

Some of the contributing factors for poor HRQOL in PWE were: un-controlled seizure, high prevalence of psychiatric disorder (depression and anxiety)[22, 23], old age, different socio-demographic factors, Poly-pharmacy [14, 17, 24–27] and being chronic disease[19]. In addition, a high rate of uncontrolled seizure in LMICs (up to 64.4%)[28–30] than high-income countries (up to 36.3%)[31] may be contributing factors for poor HRQOL in this population.

To measures the burden of the disease on daily living activity, HRQOL has been used as a measure of health indicators, apart from morbidity and mortality. But, WHO has stated,”HRQOL is the most neglected measurement in health"[32]. Thus, it should be reconsidered as one measure of treatment outcomes, in addition to seizure control [33]. Therefore, the most important goal of epilepsy treatment is now improving the patient's HRQOL[34]. However, there is a paucity of data on HRQOL of PWE living in Ethiopia. Hence, knowing the status and determinants of HRQOL is the basic step for improving HRQOL, through modification of the modifiable factors. Therefore, the aim of this study was to assess the HRQOL and its determinants among PWE.

## Methods and participants

### Study area and period

The study was conducted from April 20 /2019 –June 27/ 2019. It was done at Ambo General Hospital (AGH) which is found in Ambo town, Oromia regional state. Ambo town was located 114 km away from Addis Ababa. The Hospital has beenserving76, 774 inhabitants from the catchment area.

### Study designs

A hospital-based cross-sectional study design was used to assess the HRQOL and its determinants among adult PWE.

### Population

#### Source of population

The source population for this study was all persons living with epilepsy and who have had a follow up at AGH.

#### Study population

Adult PWE who have had follow up at AGH for at least 6 months and with inclusion criteria.

### Inclusion and exclusion criteria

**Inclusion criteria**

- Patients with Epilepsy whose age was ≥18 years.
- Patients with epilepsy having at least 6 months follow up at AGH; as criteria to evaluate for the seizure control.

**Exclusion criteria**

- Those patients who were mentally unstable such as aggressive patients, acute psychosis or panic attack or critically ill such as patients with status epileptic or any patient who is acute sick looking (determined by clinical presentation).
- Un-willing to participate in the study

### Sample size and sampling procedures

The minimum sample size that represents the general population was determined using a single population proportion formula

$$n=\left({Z_{\frac{\alpha }{2}}}^{2}\right)*\frac{P(1-P)}{W^{2}}=1.96^{2}*0.5*\frac{0.5}{0.05^{2}}=384\ldots \ldots .$$ (1)

Where n = the desirable sample size, Z (α/2) = the level of significance (1.96) at 95% C.I, p = proportion of patients’ with poor HRQOL = 0.5, W = acceptable marginal error = 0.05. Since the number of population was less than 10,000, the correction formula was used as follows:

$$nf=\frac{n}{1+\frac{n}{N}}=\frac{384}{1+\frac{384}{175}}=121+5\%(non-response)=127\ldots \ldots .$$ (2)

Where, nf = final sample size, N = the size of the population that the sample is to represent.

AGH has one Clinic in-common for patients with psychiatry and Epilepsy. The Clinic has been providing service throughout the 5 working days of the week for the patients with epilepsy. All eligible study participants; who have been visiting the clinic during the data collection period were recruited continuously until the desired sample size was achieved (consecutive sampling technique).

### Data collection tools and procedure

The data collection tool has six parts. Part I (Socio-demographic characteristics of the study participants), Part II &V (medication and clinical characteristics of the study participants), Part III (WHOQOL-BREF), Part IV (Epilepsy Self Management), and Part VI (Assessment of seizure control).

Data was collected using a validated structured questionnaire; the World Health Organization’s Quality of Life questionnaire (WHO QOL BREF) English version scale [32],was used as a data abstraction tool through face to face interview (part I, III and IV) and medical chart review (part II, V and VI). Even though there was a validated tool for assessment of psychiatric co-morbidity (depression and anxiety), it was collected by reviewing of patient’s medical chart to reduce the complexity of the questionnaire.

The generic WHOQOL-BREF contains 26 items which were divided into four domains: physical health (7 items), psychological health (6 items), social relationships (3 items), environmental health (8 items), and overall perception of their health (2 items),[32]. Each individual item of the WHOQOL-BREF was scored from 1(very dissatisfied/very poor) to 5 (very satisfied/very good) for positive questions and vice versa. Steps for checking and cleaning data and computing domain scores were performed according to the instruction provided on WHO QOL BREF manual tables 3 and 4 [32]; raw scores for the domains of WHOQOL-BREF were calculated and transformed to the scale ranging from 0 to 100. Where the score of 100is the highest and 0 is the lowest HRQOL indicators. Moreover, an overall HRQOL score was computed by adding up the scores of the four domains and then dividing by four. The mean score for each domain and the total score were calculated to categorize HRQOL of PWE. Hence, subjects were categorized as having good HRQOL in WHOQOL-BREF, if the scores are greater than or equal to mean, while participants with less than the mean score were categorized as having poor HRQOL for each domain[17, 32].

### Study variables

Dependent variable: Health-Related Quality of Life among PWE

Independent variables: Age, sex, educational status, marital status, residence, occupation, monthly income, poly-pharmacy, type of seizure, treatment outcome, co-morbid condition, duration of illness.

### Data quality assurance

All contents of the questionnaire were translated to the local language; Afaan Oromo and then translated back to English by different people to ensure consistency, except the one that was used for the collection of data from the medical chart (poly-pharmacy status, seizure control status, co-morbidity, and type of seizure). In addition, it was validated by the authors. Pre-testing was done on 5% of the total sample size at AGH to check whether the tool can capture the intended objectives. Then the pre-test data were excluded from the final analysis after adjusting for the final data abstraction tool (modification of tool for psychiatric condition assessment). The data was collected by two trained 5^th^-year pharmacy students.

### Data processing and analysis

The data were coded, checked, cleaned and then entered into SPSS version 20.0 for analysis. Descriptive analysis for continuous variables was summarized by mean (SD), whereas the categorical variables were summarized by frequency and percentage. The psychometric property of the translated (Afaan Oromo version) of the WHO QOL-BREF questionnaire was tested by authors using Cronbach's alpha test value of > 0.7 as accepted internal consistency[35–37]. Construct validity was examined using the Kaiser-Meyer-Olkin (KMO) measure of sampling adequacy with the value of ≥0.5 was used as the adequacy of the sample size for exploratory factor analysis (EFA)[38]. For categorical variables with more than two response rates, the dummy tables of n-1 were created. Then multiple linear logistic regression analysis was performed to find predictors for HRQOL. The variables with variance inflation factor (VIF) of greater than 10 were deleted from analysis to avoid multi-collinearity[39].A p-value of <0.05 was considered as independent predictors.

### Ethical consideration

Ethical clearance was obtained from the Institutional Review Ethics Committee of the College of Medicine and Health Sciences of Ambo University as well as permission was received from the clinical director of AGH. Written informed consent was sought from the study participants ahead of the interview; after explaining the purpose of the study. Confidentiality was kept.

## Results

### Socio-demographic characteristics of the respondents

Among 127 enrolled study participants, 6 study participants were excluded because of 2 participants were severely mentally ill and 4 participants were unwilling to give their consent during data collection. Thus, 121 study participants were included in the final analysis which equates with a 95.3% response rate. Majority of the study participants were male 67(55.4%), had median age of 32 years, were married 58 (47.9%), were farmer 39 (32.2%), had urban residence 62 (51.2%), had primary (1–8) education57 (47.1%), and earned<500 Ethiopian birr monthly66 (54.5%) (Table 1).

**Table 1:** Distribution of participants by socio-demographic characteristics at Ambo General Hospital, Ambo, 2019 (n = 121).

Variables	Category	Frequency	Percentage (%)
**Sex**	Male	67	55.4
	Female	54	44.6
**Age**	18–35	72	59.5
	36–55	36	29.8
	>55	13	10.7
	Median age (minimum to maximum)	32 (18 to 69)	
**Marital status**	Married	58	47.9
	Single	50	41.3
	Divorced	7	5.8
	Widowed	6	5.0
**Occupation**	Governmental employed	13	10.7
	Farmer	39	32.2
	Student	34	28.1
	Business man/women	13	10.7
	Daily labor	10	8.3
	Others#	12	9.9
**Residence**	Urban	62	51.2
	Rural	59	48.8
**Educational status**	Not educated	23	19.0
	Primary	57	47.1
	Secondary	14	11.6
	College and University(tertiary)	27	22.3
**Monthly income** **(ETB)**	<500	66	54.5
	500–1500	23	19.0
	1501–2500	9	7.4
	>2500	23	19.0

Note: at the time of this paper writing 1US$ is equal to 28.5 ETB, ETB-Ethiopian birr, other#- No work, Butcher, cleaner, tailor

### Clinical characteristics of the participants

The majority of the study participants, 45.5%have had a duration of illness related to the epilepsy of >3 years. Most of the respondents, 88.4% had seizure frequency of once per day ahead of AED initiation. In addition, 52.9% of the participants had unclassified seizure as the most common seizure diagnosis, 13.2% of the participants had Poly-pharmacy, 21.5%of the participants had un-controlled seizure (irrespective of duration since they were seizure-free), and 78.1% of the study participants had uncontrolled seizure (at least 2 years of seizure freedom), (Table 2).

**Table 2:** Clinical characteristics of the study participants at AGH, Ambo, 2019(n = 121).

Variables	Category	Frequency	Percent
**Who Knows the disease they have**	All	121	100.0
**Duration of illness**	1 year	3	2.5
	2 years	16	13.2
	3 years	47	38.8
	>3 years	55	45.5
**Frequency of seizure per day before starting AED**	Once a day	107	88.4
	Twice a day	9	7.4
	More than twice a day	5	4.1
**Poly Pharmacy**	Yes	16	13.2
	No	105	86.8
**Types of Seizures**	Simple partial seizure	1	0.8
	Absence seizure	2	1.7
	GTCS	54	44.6
	Unclassified	64	52.9
**Seizure free (irrespective of duration)**	Yes	95	78.5
	No	26	21.5
**Seizure burden (at least 2 year seizure freedom)**	Controlled	35	28.9
	Uncontrolled	86	71.1

Note: AED- Anti-epileptic drug, GTCS- Generalized tonic-clonic seizure

**Fig 1:**
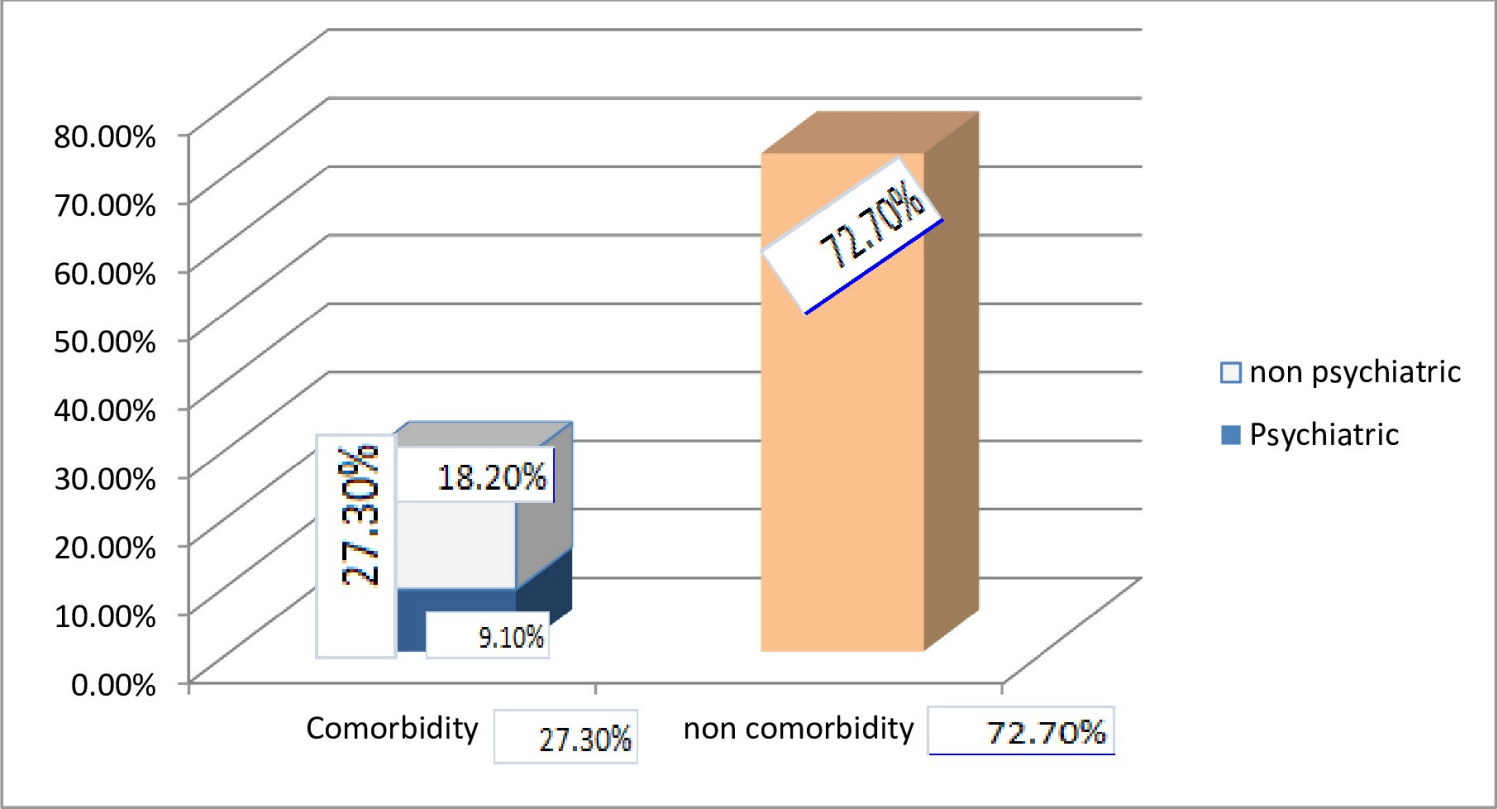
Frequency of co-morbid conditions (psychiatric and non-psychiatric) among patients with epilepsy attending AGH, Ethiopia 2019 (n = 121).

Out of the total study participants, 27.3% had co-morbid conditions. Of which 9.1% and 18.2% had psychiatric co-morbidity and non-psychiatric co-morbidity, respectively (Fig 1).

### WHOQOL-BREF (0–100) characteristics of respondents

The overall mean ±SD score using WHOQOL-BREF scale for this study was 56.42 ±10.96 and 24.4% of the study participants had overall poor HRQOL. The mean score for the environmental domain was relatively lower than the other domains while the psychological domain was higher than the rest. The validation test for the translated WHO QOL-BREF questionnaire has good internal consistency (the Cronbach’s alpha test was between 0.835 and 0.902) for each domain since it was higher than 0.7[35–37]. In addition, the KMO measure of sample size adequacy was 0.820 for the four domains’ score of HRQOL and 0.926 for the 26 items of WHO HRQOL-BREF’s facet, which indicates the appropriateness of the sample size for measuring what was intended to be measured[38],(Table 3).

**Table 3:** Distribution of HRQOL by domain using WHO HRQOL- BREF among patients with epilepsy at AGH, Ambo, Ethiopia, 2019 (n = 121).

Variables	Mean ± SD	Cronbach's Alpha	HRQOL	
			Poor (%)	Good (%)
**Physical domain(D1)**	58.42 ±13.904	.853	29(24)	92(76)
**Psychological domain(D2)**	63.42 ±13.898	.832	17(14)	104(86)
**Social domain(D3)**	51.90 ±11.185	.902	30(24.8)	91(75.2)
**Environmental domain(D4)**	51.97 ±10.791	.880	42(34.7)	79(65.3)
**Overall HRQOL**	56.43 ±10.976	.899	30(24.4)	91(75.6)

### Predictors of health-related quality of life among PWE

Multivariate linear regression analysis showed that the overall HRQOL score in PWE was negatively affected by: presence of co-morbidity (B = -5.620, SE = 1.531, p< 0.0001, 95% C.I = -8.656 to -2.584), uncontrolled seizure for at least 2 years (B = -4.239, SE = 1.296, p = 0.001, 95% C.I = -6.809 to -1.670), divorced (B = -8.423, SE = 2.241, p< 0.0001, 95%C.I = -12.867 to -3.978) relative to married PWE, and no education (B = -8.715, SE = -8.15, SE = 2.604, p = 0.001, 95%C.I = -9.648 to -1.316) relative to PWE who had level of education above tertiary. Thus, lack of seizure freedom, being divorced as compared to married, and lack of education as compared to > education level of tertiary were strong predictors for low overall HRQOL score among PWE (Table 4).

**Table 4:** Multiple linear regression analysis of variables associated with overall HRQOL among patients with epilepsy at AGH, Ambo Ethiopia, 2019 (n = 121).

Predictors for Overall HRQOL						
Variables	Unstandardized Coefficients		Standardized Coefficients	Sig.	95.0% Confidence Interval for B	
	B	Std. Error	Beta		Lower Bound	Upper Bound
**(Constant)**	69.023	4.318		<0.0001	60.459	77.588
**Age (years)**	-.016	.062	-.020	.794	-.140	.107
**Sex (reference = male)**	-.782	1.048	-.036	.457	-2.861	1.297
**place of residence (reference = urban)**	-3.281	1.514	-.150	.032	-6.284	-.279
**Co-morbidity (reference = No)**	-5.620	1.531	-.229	<0.0001	-8.656	-2.584
**poly pharmacy (reference = Yes)**	5.403	2.024	.167	.009	1.389	9.418
are you seizure free* (reference = yes)	-8.956	1.401	-.337	<0.0001	-11.736	-6.177
seizure control** (reference = yes)	-4.239	1.296	-.176	.001	-6.809	-1.670
**self management practice (reference = yes)**	.793	1.729	.025	.648	-2.637	4.222
**Single (reference = married)**	-3.808	1.641	-.172	.022	-7.063	-.552
**Divorced (reference = married)**	-8.423	2.241	-.180	<0.0001	-12.867	-3.978
**Widowed (reference = married)**	-5.058	2.394	-.100	.037	-9.806	-.310
**Unemployed (reference = yes)**	3.999	2.927	.113	.175	-1.807	9.805
**less than 1500 (reference = >2500ETB)**	-3.102	2.144	-.125	.151	-7.354	1.151
**1501 to 2500 (reference = >2500ETB)**	-.993	2.344	-.024	.673	-5.642	3.656
**no education(reference = tertiary and above)**	-8.715	2.604	-.313	.001	-13.881	-3.549
**primary education (reference = tertiary and above)**	-5.482	2.100	-.250	.010	-9.648	-1.316
**secondary education (reference = tertiary and above)**	-2.358	2.256	-.069	.298	-6.833	2.116
**greater than 2 seizure per day ahead of AED (reference = ≤2 seizure)**	1.710	2.582	.031	.509	-3.411	6.831

Note

*irrespective of seizure-free period

**at least 2 years seizure-free period, C.I- confidence interval

#### Predictors of HRQOL in physical health (domain I)

The results of multivariate linear logistic regression analysis showed that HRQOL in each domain was significantly affected by modifiable and non-modifiable factors. Accordingly, the independent predictors for low HRQOL scores of physical health were: co-morbidity, marital status, Poly-pharmacy, uncontrolled seizure, low monthly income, and low level of education. Among those factors uncontrolled seizure (irrespective of time since seizure-free) (B = -10.083, SE = 2.104, p< 0.0001, 95%C.I = -14.256 to -5.910) relative to controlled seizure, being widowed (B = -9.300, SE = 3.594, p = 0.011, 95%C.I = -16.429–2.170) relative to married and being illiterate/no education (B = -13.004, SE = 3.910, p = 0.001, 95%C.I = -20.760 to -5.248) relative to educational level of tertiary and above were found to be the strongest negative predictors of HRQOL of physical health (Table 5).

**Table 5:** Multiple linear regression analysis of variables associated with HRQOL of physical health among patients with epilepsy at AGH, Ambo Ethiopia, 2019 (n = 121).

Determinant factors for HRQOL for Domain I (Physical health)						
Explanatory variables	Unstandardized Coefficients		Standardized Coefficients	Sig.	95.0% Confidence Interval for B	
	B	Std. Error	Beta		Lower Bound	Upper Bound
**(Constant)**	66.752	6.483		<0.0001	53.892	79.612
**Age (years)**	.072	.094	.071	.444	-.114	.257
**Sex (reference = male)**	-1.567	1.574	-.056	.322	-4.688	1.555
**place of residence (reference = urban)**	-.294	2.273	-.011	.897	-4.802	4.213
**Co-morbidity (reference = No)**	-7.628	2.298	-.245	.001	-12.186	-3.069
**poly pharmacy (reference = Yes)**	7.631	3.039	.187	.014	1.603	13.659
are you seizure free* **(reference = yes)**	-10.083	2.104	-.299	<0.0001	-14.256	-5.910
seizure control** **(reference = yes)**	-5.616	1.945	-.184	.005	-9.474	-1.757
**self management practice (reference = yes)**	3.211	2.596	.079	.219	-1.938	8.361
**Single (reference = married)**	-.408	2.464	-.014	.869	-5.296	4.480
**Divorced (reference = married)**	-6.574	3.364	-.111	.053	-13.247	.099
**Widowed (reference = married)**	-9.300	3.594	-.146	.011	-16.429	-2.170
**Unemployed (reference = yes)**	5.104	4.395	.114	.248	-3.614	13.822
**less than 1500 (reference = >2500ETB)**	-6.910	3.219	-.220	.034	-13.294	-.525
**1501 to 2500 (reference = >2500ETB)**	-4.719	3.519	-.089	.183	-11.699	2.262
**no education (reference = tertiary and above)**	-13.004	3.910	-.368	.001	-20.760	-5.248
**primary education (reference = tertiary and above)**	-8.518	3.154	-.307	.008	-14.773	-2.263
**secondary education (reference = tertiary and above)**	-4.333	3.387	-.100	.204	-11.052	2.385
**greater than 2 seizure per day ahead of AED (reference = ≤2 seizure)**	2.917	3.876	.042	.453	-4.772	10.606

Note

*irrespective of seizure-free period

**at least 2 years seizure-free period, C.I- confidence interval

#### Predictors of HRQOL of psychological health (domain II)

Furthermore, presence of co-morbidity, rural residence, lack of seizure control, and being divorced were negative predictors (all p< 0.05) for HRQOL score of psychological health. Indeed, uncontrolled seizure (irrespective of time since seizure-free) (B = -12.668, SE = 2.019, p< 0.0001, 95%C.I = -16.671 to -8.664) compared to controlled seizure and being divorced (B = -10.153, SE = 3.228, p = 0.002, 95%C.I = -16.556 to -3.751) compared to married were strong predictors for low HRQOL score of psychological health (Table 6).

**Table 6:** Multiple linear regression analysis of variables associated with HRQOL of psychological health among patients with epilepsy at AGH, Ambo Ethiopia, 2019 (n = 121).

Determinant factors for HRQOL for Domain II (Psychological health)						
Variables	Unstandardized Coefficients		Standardized Coefficients	Sig.	95.0% Confidence Interval for B	
	B	Std. Error	Beta		Lower Bound	Upper Bound
**(Constant)**	81.088	6.220		<0.0001	68.751	93.426
**Age (years)**	-.016	.090	-.016	.859	-.194	.162
**Sex (reference = male)**	1.778	1.510	.064	.242	-1.217	4.772
**place of residence (reference = urban)**	-5.363	2.180	-.194	.016	-9.687	-1.038
**Co-morbidity (reference = No)**	-7.321	2.205	-.236	.001	-11.694	-2.947
**poly pharmacy (reference = Yes)**	4.876	2.916	.119	.097	-.907	10.659
are you seizure free* **(reference = yes)**	-12.668	2.019	-.376	<0.0001	-16.671	-8.664
seizure control**(reference = yes)	-3.960	1.866	-.130	.036	-7.662	-.259
**self management practice (reference = yes)**	-2.109	2.491	-.052	.399	-7.049	2.832
**Single (reference = married)**	-2.240	2.364	-.080	.346	-6.929	2.450
**Divorced (reference = married)**	-10.153	3.228	-.171	.002	-16.556	-3.751
**Widowed (reference = married)**	-4.496	3.448	-.071	.195	-11.336	2.344
**Unemployed (reference = yes)**	1.811	4.217	.041	.668	-6.553	10.175
**less than 1500 (reference = >2500ETB)**	-1.134	3.088	-.036	.714	-7.259	4.992
**1501 to 2500 (reference = >2500ETB)**	-2.290	3.376	-.043	.499	-8.987	4.406
**no education (reference = tertiary and above)**	-6.911	3.751	-.196	.068	-14.352	.530
**primary education (reference = tertiary and above)**	-3.198	3.025	-.115	.293	-9.199	2.803
**secondary education (reference = tertiary and above)**	-.115	3.250	-.003	.972	-6.561	6.331
**greater than 2 seizure per day ahead of AED (reference = ≤2 seizure)**	-3.210	3.719	-.046	.390	-10.587	4.167

Note

*irrespective of seizure-free period

**at least 2 years seizure-free period, C.I- confidence interval

#### Predictors of HRQOL of social health (domain III)

Low HRQOL score of social health was significantly associated with marital status, Poly-pharmacy, uncontrolled seizure & lower educational level (all p< 0.05). Of this explanatory variables, absence of Poly-pharmacy (B = 9.050, SE = 3.027, P = 0.003, 95%C.I = 3.047 to 15.054) relative to presence of poly-pharmacy, being single (B = -9.551, SE = 2.095, p< 0.0001, 95%C.I = -14.419 to -4.683), and divorced (B = -11.022, SE = 3.351, P = 0.001, 95%C.I = -17.668 to -4.376) relative to married were found to be strong independent predictors for HRQOL score of social health among PWE (Table 7).

**Table 7:** Multiple linear regression analysis of variables associated with HRQOL of social health among patients with epilepsy at AGH, Ambo Ethiopia, 2019 (n = 121).

Determinant factors for Domain III (Social health)						
Variables	Unstandardized Coefficients		Standardized Coefficients	Sig.	95.0% Confidence Interval for B	
	B	Std. Error	Beta		Lower Bound	Upper Bound
**(Constant)**	57.607	6.457		<0.0001	44.800	70.415
**Age (years)**	-.179	.093	-.220	.058	-.364	.006
**Sex (reference = male)**	-2.847	1.567	-.127	.072	-5.956	.261
**place of residence (reference = urban)**	-1.674	2.263	-.075	.461	-6.163	2.816
**Co-morbidity (reference = No)**	-3.301	2.289	-.132	.152	-7.841	1.239
**poly pharmacy (reference = Yes)**	9.050	3.027	.275	.003	3.047	15.054
are you seizure free* **(reference = yes)**	-6.167	2.095	-.227	.004	-10.324	-2.011
seizure control** **(reference = yes)**	-2.861	1.937	-.116	.143	-6.704	.981
**self management practice (reference = yes)**	3.198	2.586	.097	.219	-1.931	8.326
**Single (reference = married)**	-9.551	2.454	-.422	<0.0001	-14.419	-4.683
**Divorced (reference = married)**	-11.022	3.351	-.231	.001	-17.668	-4.376
**Widowed (reference = married)**	-6.575	3.580	-.128	.069	-13.675	.526
**Unemployed (reference = yes)**	9.044	4.377	.251	.041	.362	17.726
**less than 1500 (reference = >2500ETB)**	-2.768	3.206	-.110	.390	-9.126	3.591
**1501 to 2500 (reference = >2500ETB)**	3.069	3.505	.072	.383	-3.883	10.021
**no education (reference = tertiary and above)**	-8.590	3.894	-.303	.030	-16.314	-.866
**primary education (reference = tertiary and above)**	-6.871	3.141	-.308	.031	-13.100	-.641
**secondary education (reference = tertiary and above)**	-6.758	3.373	-.194	.048	-13.450	-.067
**greater than 2 seizure per day ahead of AED (reference = ≤2 seizure)**	4.626	3.861	.083	.234	-3.031	12.284

Note

*irrespective of seizure-free period

**at least 2 years seizure-free period, C.I- confidence interval

#### Predictors of HRQOL of environmental health (domain IV)

Low HRQOL score of environmental health was independently predicted by palace of residence (B = -5.795, SE = 2.101, p = 0.007, 95%C>I = -9.962 to -1.628), co-morbidity (B+ -4.230, SE = 2.125, p = 0.049, 95%C.I = -8.444 to -0.015) & uncontrolled seizure irrespective of time since seizure-free (B = -6.907, SE = 1.945, p = 0.001, 95%C.I = -10.765 to -3.049) and uncontrolled seizure of at least 2 years (B = -4.520, SE = 1.798, p = 0.014, 95%C.I = -8.088 to -0.953), (Table 8).

**Table 8:** Multiple linear regression analysis of variables associated with HRQOL of environmental health among patients with epilepsy at AGH, Ambo Ethiopia, 2019 (n = 121).

Determinant factors for Domain IV (Environmental health)						
Variables	Unstandardized Coefficients		Standardized Coefficients	Sig.	95.0% Confidence Interval for B	
	B	Std. Error	Beta		Lower Bound	Upper Bound
**(Constant)**	70.645	5.994		<0.0001	58.756	82.534
**Age (years)**	.058	.086	.073	.506	-.114	.229
**Sex (reference = male)**	-.492	1.455	-.023	.736	-3.378	2.393
**place of residence (reference = urban)**	-5.795	2.101	-.270	.007	-9.962	-1.628
**Co-morbidity (reference = No)**	-4.230	2.125	-.175	.049	-8.444	-.015
**poly pharmacy (reference = Yes)**	.055	2.810	.002	.984	-5.517	5.628
are you seizure free* **(reference = yes)**	-6.907	1.945	-.264	.001	-10.765	-3.049
seizure control** **(reference = yes)**	-4.520	1.798	-.191	.014	-8.088	-.953
**self management practice (reference = yes)**	-1.130	2.400	-.036	.639	-5.891	3.630
**Single (reference = married)**	-3.032	2.278	-.139	.186	-7.551	1.486
**Divorced (reference = married)**	-5.942	3.110	-.129	.059	-12.111	.228
**Widowed (reference = married)**	.138	3.323	.003	.967	-6.453	6.730
**Unemployed (reference = yes)**	.038	4.063	.001	.993	-8.022	8.097
**less than 1500 (reference = >2500ETB)**	-1.596	2.976	-.066	.593	-7.499	4.307
**1501 to 2500 (reference = >2500ETB)**	-.033	3.253	-.001	.992	-6.486	6.420
**no education (reference = tertiary and above)**	-6.355	3.615	-.232	.082	-13.526	.815
**primary education (reference = tertiary and above)**	-3.341	2.915	-.155	.255	-9.124	2.442
**secondary education (reference = tertiary and above)**	1.773	3.131	.053	.572	-4.438	7.985
**greater than 2 seizure per day ahead of AED (reference = ≤2 seizure)**	2.508	3.584	.046	.486	-4.601	9.616

Note

*irrespective of seizure-free period

**at least 2 years seizure-free period, C.I- confidence interval

## Discussion

This study sought to investigate the HRQOL among PWE using WHO HRQOL-BREF and its determinants because HRQOL has been used for health measurement, in addition to morbidity and mortality in patients with epilepsy[32].

Overall poor HRQOL was recorded in 24.4% of the study participants, which is lower than the study conducted at Amanuel Mental Specialized Hospital (AMSH) 45.8% of poor HRQOL[17]. This difference may be partially explained by the high prevalence of anxiety (33.5%) and depression (32.8%) in the latter study versus 9.1% of psychiatric co-morbidity because psychiatric co-morbidity was an independent predictor for poor HRQOL[17, 40]. The prevalence of poor HRQOL among PWE in this study varies between the domains. In our study, among the four domains better mean HRQOL score was achieved in the psychological domain (63.42 ±13.898). It is congruent with the study done in India [24]. In contrast, a better mean of HRQOL scores was reported in the social domain by another study[20]. This indicates that there was an inconsistent mean score of HRQOL throughout the world owing to different reasons. Indeed, for our study, the mean HRQOL score for each domain was between 51.90 and 63.42, which was slightly lower than the study done in Iran (59.05 to 78.43)[37]. In addition, our study found that a lower mean HRQOL score was recorded in environmental and social domains. This finding was in line with the study finding from AMSH, Ethiopia[17].

The mean overall HRQOL score in this study was 56.4±12.4 which was higher than the study conducted at Tamil Nadu, India (51.49)[20]. However, the score was lower than another study done in India (63.05)[24] as well as two studies from Iran (67.01)[37] and 68.03[36]. Perhaps, the possible reasons for the differences may be (1)due to variation in the percentage of co-morbidity (India-13% and 27%) which may contribute for low mean HRQOL score in our study finding,(2) due to difference in the tool (WHO QOL-BREF and QOLIE-31), (3) due to difference in the study population (general adult PWE and female PWE).

The predictors for HRQOL among PWE was inconsistent[40], indeed, overall HRQOL score was significantly dragged down by the presence of co-morbidity[17, 40], uncontrolled seizure for at least 2 years[17, 24, 40], being divorced and low level of education according to our study analysis result[17, 24, 40].

Lower HRQOL score of the physical domain was significantly contributed by the presence of co-morbidity, divorced and widowed [24], Poly-pharmacy[40], uncontrolled seizure, low level of monthly income[24, 37, 40], and low level of education[17, 24, 40]. However, the study conducted in Bulgaria showed that there was no significant association between low HRQOL score and marital status[41]. The possible reasons for the differences might be, attributed to generic WHOQOL-BREF use, unlike that of the study done in Bulgaria which used the Quality of Life Inventory for Epilepsy (HRQOLIE-89). Indeed, there is a difference between the tools in exploring the detail factor.

On top of this, our study revealed that co-morbidity, rural residence, uncontrolled seizure[40], and being divorced were an independent predictor for low HRQOL score of psychological health in PWE, which was in line with different study findings[22, 42]. In particular, psychiatric co-morbidities (depression and anxiety) were tuned out to be the independent predictors for low HRQOL in this domain[17, 23, 43];negatively affects psychological HRQOL in PWE [42].

The current study disclosed that being single or divorced, uncontrolled seizure & low level of education [14, 17, 26, 27, 43], and poly-pharmacy were independent predictors for low HRQOL scores of social health. This finding was congruent with previous studies [14, 24]. Besides this, patients who had Poly-pharmacy might have adverse effects and un-affordability issue which can negatively affect the HRQOL score[27, 44].

Furthermore, our study revealed that rural residence, co-morbidity[24] and uncontrolled seizure were found to be significant predictors for low HRQOL scores of environmental health. The current result was in agreement with the different studies [14, 26, 27, 43]. Thus, effectively controlling the burden of seizure attack is paramount to increase the HRQOL score for PWE. Especially, in LMICs, like that of Ethiopia where the prevalence of uncontrolled seizure was high (64.4%) owing to different factors [28–30] than high-income countries [31];which hijacks the life of the patients and their family as a whole.

The study was not without limitation; some of the patient’s charts were incomplete due to loss of the initial chart and/or transfer-in from other hospitals; that may affect the quality of collected data; for which we asked the patients for their seizure-free period (recall bias). In addition being single-center, which might not represent the HRQOL of PWE in Ethiopia that limits the broader applications of the findings. Finally, the generic WHO HRQOL-BREF is not good enough to disclose the determinants of HRQOL unlike that of epilepsy specify tools, despite the fact that it was validated with good internal consistency and reliability (α>0.7& KMO>0.5).

## Conclusion and recommendation

The prevalence of poor health-related quality of life among PWE was almost within the range of reports by other studies. In general, a low level of HRQOL score was significantly associated with the marital status of single/widowed/divorced, low level of education, low level of monthly income, co-morbidity, uncontrolled seizure, and poly-pharmacy, irrespective of specific HRQOL domain.

Thus, West Shoa zone health bureau, AGH, and clinicians should work on the modifiable factors to improve the HRQOL score for PWE as the treatment of epilepsy alone does not suffices to improve epilepsy treatment outcomes. Furthermore, they should have to work on improving seizure control, detecting and treating psychiatric or other co-morbid illness, and try to reduce Poly-pharmacy, as they had contributed to poor HRQOL in PWE. Further studies are required to evaluate HRQOL in epilepsy with a multicenter study design using epilepsy specific tools to explore for HRQOL improvement methods as one goal of epilepsy treatment.

## Supporting information

S1 File Data abstraction tool for HRQOL. (SAV)

S2 File SPSS dataset for PLOS ONE HRQOL. (DOCX)

## Abbreviations

AEDs: Antiepileptic drugs
AGH: Ambo General Hospital
HRQOL: Health-Related Quality of Life.
ILAE: International League against Epilepsy
LMIC: Low and Middle-income country
KMO: Kaiser-Meyer-Olkin
PWE: Patients with epilepsy
WHO: World Health Organization
WHOQOL BREF: World Health Organization Quality of Life BREF Questionnaire
