# Supplementary material for: Health-related quality of life and its determinants among ambulatory patients with epilepsy at Ambo General Hospital, Ethiopia: Using WHOQOL-BREF
Source: PLoS One. 2020 Jan 21;15(1):e0227858. doi: 10.1371/journal.pone.0227858 (PMC6974038; doi:10.1371/journal.pone.0227858)
Supplement: S2 File — (DOCX) [file pone.0227858.s002.docx]

## S1 data abstraction tool for HRQOL (English version of questionnaire)

**Part I.** **Socio-demographic characteristics of the study participants.**

1. Card number______________________

2. Age _______Years

3. Sex a) Male [ ] b) Female [ ]

4. Ethnicity a) Oromo [ ] b) Amhara [ ] c) Tigre [ ]

d) Gurage [ ] e) Somali[ ] f) Others, specify-----------------------------------

5. Religion a) Muslim [ ] b) Orthodox [ ] c) Protestant [ ] d) Others [ ]specify---------------------------------

6. Marital status (a) Married [ ] (b) Single [ ] (c) Divorced [ ] (d) Widowed [ ]

7. Occupation (a) government employee [ ] (b) farmer [ ] (c) Student [ ]

(d) Businessman/woman [ ] (e) daily labor [ ] (f) others specify ………………….

8. Place of residence (a) urban [ ] (b) rural [ ]

9. Level of education (a) Not educated [ ] (b) Primary(1-8) [ ] (C) Secondary (8-12) [ ] (d) Colleges and university [ ]

10. How much is your monthly income? (a)<500 [ ] (b) 500-1000 [ ] (c) 1000-2000 [ ] (d) >2000 [ ]

**Part II : disease condition**

a) Do you know the disease you have? YES NO

b) Have you had this disease before? YES NO

c) For how long do you have stayed with this disease? 1 year 2 years 3 years above

d) How many time do you experienced with this symptoms /day. Once a day Twice a day

more than two

**Part III. WHOQOL-BREF**

Please keep in mind your standards, hopes, pleasures and concerns. I ask that you think about your life in the last four weeks (The overall quality of life and general health facet).

|  |  | Very poor | Poor | Neither poor nor good | Good | Very good |
| --- | --- | --- | --- | --- | --- | --- |
| 1 | How would you rate your quality of life? | 1 | 2 | 3 | 4 | 5 |
|  |  | Very dissatisfied | Dissatisfied | Neither satisfied nor dissatisfied | Satisfied | Very satisfied |
| 2 | How satisfied are you with your health? | 1 | 2 | 3 | 4 | 5 |

The following questions ask about ***how much*** you have experienced certain things in the last two weeks.

|  |  | Not at all | A little | A moderate amount | Very much | An extreme amount |
| --- | --- | --- | --- | --- | --- | --- |
| 3 | To what extent do you feel that physical pain prevents you from doing what you need to do? | 1 | 2 | 3 | 4 | 5 |
| 4 | How much do you need any medical treatment to function in your daily life? | 1 | 2 | 3 | 4 | 5 |
| 5 | How much do you enjoy life? | 1 | 2 | 3 | 4 | 5 |
| 6 | To what extent do you feel your life to be meaningful? | 1 | 2 | 3 | 4 | 5 |
|  |  | Not at all | A little | A moderate amount | Very much | Extremely |
| 7 | How well are you able to concentrate? | 1 | 2 | 3 | 4 | 5 |
| 8 | How safe do you feel in your daily life? | 1 | 2 | 3 | 4 | 5 |
| 9 | How healthy is your physical environment? | 1 | 2 | 3 | 4 | 5 |

The following questions ask about ***how completely*** you experience or were able to do certain things in the last two weeks.

|  |  | Not at all | A little | Moderately | Mostly | Completely |
| --- | --- | --- | --- | --- | --- | --- |
| 10 | Do you have enough energy for everyday life? | 1 | 2 | 3 | 4 | 5 |
| 11 | Are you able to accept your bodily appearance? | 1 | 2 | 3 | 4 | 5 |
| 12 | Have you enough money to meet your needs? | 1 | 2 | 3 | 4 | 5 |
| 13 | How available to you is the information that you need in your day-to-day life? | 1 | 2 | 3 | 4 | 5 |
| 14 | To what extent do you have the opportunity for leisure activities? | 1 | 2 | 3 | 4 | 5 |

|  |  | Very poor | Poor | Neither poor nor good | Good | Very good |
| --- | --- | --- | --- | --- | --- | --- |
| 15 | How well are you able to get around? | 1 | 2 | 3 | 4 | 5 |

The following questions ask you to say ***how good or satisfied*** you have felt about various aspects of your life over the last two weeks**.**

|  | |  | Very dissatisfied | Dissatisfied | Neither satisfied nor dissatisfied | Satisfied | Very satisfied |
| --- | --- | --- | --- | --- | --- | --- | --- |
| 16 | | How satisfied are you with your sleep? | 1 | 2 | 3 | 4 | 5 |
| 17 | | How satisfied are you with your ability to perform your daily living activities? | 1 | 2 | 3 | 4 | 5 |
| 18 | | How satisfied are you with your capacity for work? | 1 | 2 | 3 | 4 | 5 |
| 19 | | How satisfied are you with yourself? | 1 | 2 | 3 | 4 | 5 |
| 20 | | How satisfied are you with your personal relationships? | 1 | 2 | 3 | 4 | 5 |
| 21 | | How satisfied are you with your sex life? | 1 | 2 | 3 | 4 | 5 |
| 22 | | How satisfied are you with the support you get from your friends? | 1 | 2 | 3 | 4 | 5 |
| 23 | | How satisfied are you with the conditions of your living place? | 1 | 2 | 3 | 4 | 5 |
| 24 | | How satisfied are you with your access to health services? | 1 | 2 | 3 | 4 | 5 |
| 25 | How satisfied are you with your transport? | | 1 | 2 | 3 | 4 | 5 |

The following question refers to ***how often*** you have felt or experienced certain things in the last two weeks.

|  |  | Never | Seldom | Quite often | Very often | Always |
| --- | --- | --- | --- | --- | --- | --- |
| 26 | How often do you have negative feelings such as blue mood, despair, anxiety, depression? | 1 | 2 | 3 | 4 | 5 |

**Part IV Epilepsy Self Management (Practitioner)**

Evaluation Form for Epilepsy Self Management Protocol (Practitioner) Please circle one: Physician Nurse Practitioner Nurse

Please answer the following with an **X** in the yes or no box:

|  | Yes | No |
| --- | --- | --- |
| Were the Self management patient packets easy to find? |  |  |
| Usability of educational information |  |  |
| Information was easy to understand |  |  |
| Is the information timely and applicable to epilepsy  Patients |  |  |
| Comments |  |  |

**Epilepsy Self Management Protocol Evaluation Form (Patients)**

**1.** Please Answer the Following by marking an **X** in the yes or no box below

|  | Yes | No |
| --- | --- | --- |
| Did you receive information about seizure medications and possible side effects? |  |  |
| Did you receive information describing the type of seizures you have? |  |  |
| Did you receive information about safety issues related to seizures, such as no driving until seizure free for 6 months? |  |  |
| Was the information you were given by the nurses easy to read and understand? |  |  |

**2**. Please Evaluate the Following by marking an **X** in the yes or no box below

|  | Yes | No |
| --- | --- | --- |
| Did you visit the Epilepsy web sites? |  |  |
| Did you find the web site information useful? |  |  |
| Was the educational information you received about your seizure type, medication, safety issue helpful? |  |  |
| Would you recommend the Epilepsy Self Management Education Session be given to all patients? |  |  |

**Part V: Assessment of co morbid condition from the patient’s medical records**

1. Is there any co morbid condition? A. yes B. no

2. If yes what types of co morbid are there?

A. psychiatric co morbidity B. other condition specify………

3. Is there poly pharmacy? a) Yes b) no

4. Types of seizure ……………

**VII. Tools for Assessing Seizure control**

1. Are you seizure free? A. Yes B. No

2. If the above question is yes, for how long you are free from seizure?

A. > 2 years B. 1-2 years C. 6 months to 1 year D. < 06 months
